# The Hippo Signaling Pathway Manipulates Cellular Senescence

**DOI:** 10.3390/cells14010013

**Published:** 2024-12-26

**Authors:** Chiharu Miyajima, Mai Nagasaka, Hiromasa Aoki, Kohki Toriuchi, Shogo Yamanaka, Sakura Hashiguchi, Daisuke Morishita, Mineyoshi Aoyama, Hidetoshi Hayashi, Yasumichi Inoue

**Affiliations:** 1 Department of Cell Signaling, Graduate School of Pharmaceutical Sciences, Nagoya City University, Nagoya 467-8603, Japan; mai.nagasaka@jfcr.or.jp (M.N.); c232740@ed.nagoya-cu.ac.jp (S.Y.); c232732@ed.nagoya-cu.ac.jp (S.H.); daisuke.morishita@chordiatherapeutics.com (D.M.); hhayashi@phar.nagoya-cu.ac.jp (H.H.); 2 Department of Experimental Chemotherapy, Cancer Chemotherapy Center of JFCR, Tokyo 135-8550, Japan; 3 Department of Pathobiology, Graduate School of Pharmaceutical Sciences, Nagoya City University, Nagoya 467-8603, Japan; haoki@phar.nagoya-cu.ac.jp (H.A.); ko-tori@phar.nagoya-cu.ac.jp (K.T.); aomine@phar.nagoya-cu.ac.jp (M.A.)

**Keywords:** Hippo pathway, cellular senescence, cancer

## Abstract

The Hippo pathway, a kinase cascade, coordinates with many intracellular signals and mediates the regulation of the activities of various downstream transcription factors and their coactivators to maintain homeostasis. Therefore, the aberrant activation of the Hippo pathway and its associated molecules imposes significant stress on tissues and cells, leading to cancer, immune disorders, and a number of diseases. Cellular senescence, the mechanism by which cells counteract stress, prevents cells from unnecessary damage and leads to sustained cell cycle arrest. It acts as a powerful defense mechanism against normal organ development and aging-related diseases. On the other hand, the accumulation of senescent cells without their proper removal contributes to the development or worsening of cancer and age-related diseases. A correlation was recently reported between the Hippo pathway and cellular senescence, which preserves tissue homeostasis. This review is the first to describe the close relationship between aging and the Hippo pathway, and provides insights into the mechanisms of aging and the development of age-related diseases. In addition, it describes advanced findings that may lead to the development of tissue regeneration therapies and drugs targeting rejuvenation.

## 1. Introduction

The Hippo pathway, which regulates cell differentiation and proliferation, is an evolutionarily conserved signaling cascade that is regulated by a wide range of signaling pathways to control tissue growth and cell fate [1,2,3]. This pathway was discovered by the identification of Wts [4,5] and Hpo [6,7], kinases that regulate organ size and cell overgrowth in *Drosophila*. In mammals, an upstream stimulation of mammalian sterile 20-like kinase 1/2 (MST1/2), a homolog of *Drosophila* Hpo, was shown to activate the normal Hippo signaling pathway. MST1/2 forms a complex with Salvador family WW domain-containing protein 1 (SAV1) and Mps one binder kinase activator-like 1A and 1B to phosphorylate and activates the serine/threonine kinase large tumor suppressor 1/2 (LATS1/2). LATS1/2 binds to the transcriptional co-activator Yes-associated protein (YAP) and its paralog WW domain-containing transcriptional regulator 1 (TAZ). The phosphorylation of YAP/TAZ promotes their binding to 14-3-3 and either retains TAZ/YAP in the cytosol or degrades them via proteasomes [8,9,10,11]. Therefore, YAP/TAZ cannot enter the nucleus, and their interaction with transcription factors, such as TEA domain (TEAD) family members, is repressed, which negatively regulates the expression of downstream gene targets [12,13,14]. A dysfunction in this core kinase cascade leads to a hyperproliferative phenotype, causing abnormal cell proliferation and organ enlargement [15]. The activities of these kinases and scaffold components are involved in homeostasis in various tissues and carcinogenesis [16]. Screening for target factors regulated by YAP/TAZ and TEAD recently provided novel insights into the physiological functions of the Hippo pathway [17,18]. This review focuses on the impact of the Hippo pathway on cellular senescence in the human body and provides an overview of causes ranging from physiological processes, such as embryonic development, tissue remodeling, and tissue repair, to disease mechanisms.

There is a limit to the number of times a normal human somatic cell divides, and the cellular changes that progress toward this division limit are called “cellular senescence”. Cellular senescence is a mechanism for the irreversible arrest of cell proliferation and was first described by Hayflick et al. in 1961 [19]. This phenomenon is caused by the gradual shortening of telomeres through repeated cell division and was identified as a physiological response that prevents genomic instability and the accumulation of DNA damage. In 1986, Smith et al. reported that senescent cells highly expressed cellular senescence-inducing genes that arrest cell division [20]. Furthermore, homeostatic DNA damage responses (DDRs), such as oxidative stress, radiation, and the activation of oncogenes, may cause the arrest of cell division, similar to cellular senescence [21]. DDRs induce cellular senescence by arresting the cell cycle via the activation of DDR signaling kinases and p53. The phenotypes of senescence include the increased expression of the cell cycle regulators p16/p21, oxidative damage due to increased reactive oxygen species (ROS) levels, and the induced expression of BCL2, an anti-apoptotic factor. The induction of cellular senescence inhibits the regenerative capacity of tissues due to impaired stem cell differentiation and proliferation, and causes chronic inflammation due to the secretion of the senescence-associated secretory phenotype (SASP) factor [22]. Furthermore, senescence occurs in various tumors in an organism and has been shown to halt tumor development and progression. On the other hand, cultured cells in which cellular senescence has been induced by various stresses contain secreted factors that promote the malignant transformation of cancer [23,24]. This secretory phenomenon, which includes inflammatory cytokines, chemokines, collagenases, and growth factors, is called SASP and is being increasingly researched [25,26,27]. The accumulation of senescent cells is known to occur in several age-related diseases, and strategies to exploit senescent cells for treatment are attracting attention. Recent advances have been achieved in the development and preclinical/clinical applications of agents that selectively remove senescent cells and also in our understanding of the effects of deleterious senescent cell secretions (SASPs) for the treatment of diseases. Senescent cells cause inflammatory diseases, and age-related conditions, such as osteoarthritis (OA) [28] and atherosclerosis [29], and dysfunctions in normal tissue function via SASP. Since cellular senescence may act in an inhibitory or promotive manner in cancer, the complex mechanisms involved need to be elucidated in more detail. This review describes the crosstalk between the Hippo pathway and cellular senescence and focuses on its impact on disease and potential therapeutic applications.

## 2. Senescence and the Hippo Pathway

**Figure 1:**
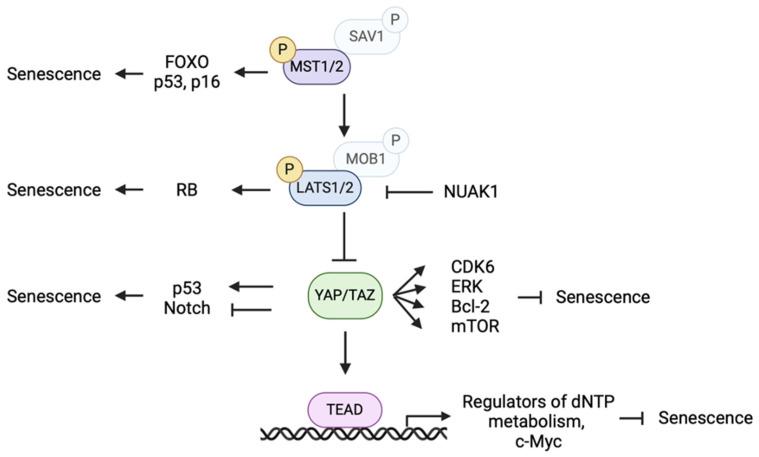
Effects of the Hippo pathway on cellular senescence [30,31,34,35,37,38,40,44,47,51,53]. Factors associated with the Hippo pathway contribute to cellular senescence by regulating a number of molecules. MST1/2 and LATS1/2 promote senescence, whereas YAP/TAZ promote and inhibit senescence depending on the molecules associated. TEAD contributes to the expression of molecules that inhibit senescence. Arrows and perpendicular bars indicate potentiating and inhibitory effects.

Key molecules in the Hippo pathway play an important role in the regulation of cellular senescence. In this Section, we describe the contribution of Hippo pathway-related molecules to cellular senescence (Figure 1).

### 2.1. YAP/TAZ

In the Hippo pathway, well-known transcriptional coactivators that activate transcription are YAP and its paralog, TAZ. They play a major role in organ size regulation, cell proliferation, and tumorigenesis, which are regulated by the Hippo pathway. Recent studies reported that YAP transcriptionally regulated *cyclin D-dependent kinase 6* (*CDK6*) gene expression and was crucial for maintaining normal cell growth and suppressing cellular senescence [30]. The anti-senescence function of YAP/CDK6 plays a pro-carcinogenic role in tumor cells. Melanoma cell proliferation is strongly dependent on the CDK4/6-mediated inhibition of cellular senescence. CDK4/6 was found to positively regulate the cell cycle by stabilizing and activating forkhead box M1, suppressing ROS levels, and protecting cancer cells from senescence [31]. Cancer cell growth with the activation of CDK4/6 may be dependent on YAP; therefore, the inhibition of YAP may provide new therapeutic tools for insights into liver cancer and melanoma.

Yippee-like 3 (*YPEL3*), a target gene of p53, has been shown to reduce tumor malignancy by inducing cellular senescence. YAP inhibits cellular senescence by suppressing YPEL3 expression in breast cancer cells [32]. The inhibitory role of YAP in the regulation of YPEL3-induced oxidative stress and apoptosis suggests that the Hippo pathway is crucial for the regulation of cellular senescence.

Aging is closely related to the induction of cellular senescence. In stromal cells, YAP/TAZ activity was shown to decrease with physiological aging, which induced structural and functional declines in aging tissues [33]. Furthermore, the inactivation of YAP/TAZ increased the number of senescent cells in vivo and accelerated the appearance of age-related tissue degeneration because the mechanotransduction of YAP/TAZ suppressed cGAS-STING signaling, a classical innate immune response pathway. YAP/TAZ activity has been suggested to suppress immunity and manipulate inflammation associated with aging, leading to healthy aging control. Further studies on the molecular mechanisms that regulate cellular senescence and aging, with a focus on the YAP/TAZ-cGAS-STING signaling pathway, will clarify the mechanisms responsible for age-related diseases, such as cancer and inflammatory diseases.

YAP/TAZ are also suppressively involved in stem cell senescence. Stem cell senescence inhibits the supply of cells to tissues, leading to the development of diseases caused by aging. In human periodontal ligament stem cells, YAP regulated the ERK and Bcl-2 signaling pathways to inhibit apoptosis and delay cellular senescence [34]. Previous studies demonstrated the involvement of YAP/TAZ in stem cell maintenance [35,36,37]. YAP/TAZ act via mTOR in stem cell proliferation and maintenance [35]. In epidermal stem cells, the activation of YAP/TAZ maintained stem cells by inhibiting Notch signaling, a key factor in epidermal differentiation [37]. Moreover, the inhibition of YAP effectively induced the differentiation of functional insulin-producing β-cells from stem cells, whereas its expression maintained stem cells [36]. Collectively, these findings suggest that YAP/TAZ regulate stem cell senescence through a number of signals.

A previous study reported that the excessive activation of YAP/TAZ induced p53-dependent cellular senescence and apoptosis in hepatocytes [38]. The excessive activation of YAP/TAZ induces p53 activation by causing DNA damage, and p53 induces p21 expression and CHK2 phosphorylation levels, thereby inducing cellular senescence. YAP/TAZ have been suggested to regulate cell fates through their control of TGF-β signaling and Hnf4a expression during liver development. Depending on the degree of cell differentiation, the molecules regulated by YAP/TAZ expression may also change, and their effects on cellular senescence may be positive or negative. Therefore, the role of YAP/TAZ in cellular senescence needs to be examined in more detail by investigating its molecular expression in cancer cells.

### 2.2. TEAD

The TEAD family of transcription factors binds to and regulates transcription with YAP/TAZ, which lack a DNA-binding domain. Although YAP/TAZ interact with a number of transcription factors, they have been shown to primarily utilize TEADs when involved in biologically important gene expression [39]. Mammals have four highly conserved TEADs that bind to the same DNA sequences; however, they perform different functions during development. The unique regulation of senescence by the YAP/TAZ-TEAD complex has been reported.

TEADs, in combination with YAP/TAZ, regulate the transcription of enzymes involved in deoxynucleotide (dNTP) biosynthesis [40]. This complex directly supports DNA replication by maintaining intracellular dNTP levels. Furthermore, YAP-TEAD overcomes the Ras-induced inhibition of dNTP metabolism and senescence by regulating dNTP metabolism. These findings indicate that YAP/TAZ-TEAD links cell proliferation with metabolism that contributes to DNA replication and promotes escape from oncogene-induced senescence.

The YAP-TEAD complex is involved in the survival of senescent cells through its regulation of the biosynthetic function of the endoplasmic reticulum (ER). The ER of senescent cells is under stress due to the active synthesis of proteins required for the execution of SASP [41]. Therefore, if the biosynthetic capacity of the ER of senescent cells is reduced, they will die by apoptosis as a result of a metabolic crisis. In the aged human diploid fibroblast cell line, WI-38, the expression of genes in the YAP-TEAD1/2 pathway, particularly TEAD2, was shown to play a role in the viability of senescent cells [42]. YAP-TEAD leads senescent cells to survival while controlling ER stress through mTOR functions. Verteporfin [43], which inhibits the YAP-TEAD interaction, has been shown to induce apoptosis in senescent cells and, thus, the suppression of this pathway is expected to lead to the selective elimination of senescent cells. Furthermore, the YAP/TEAD complex has been suggested to promote cell transformation via the regulation of c-Myc expression, leading to tumorigenesis [44]. TEAD4 is known to form a complex with YAP/TAZ in order to induce the expression of target genes involved in promoting the cell cycle, which results in cell transformation to cancer [45]. Since YAP/TAZ/TEAD-c-Myc may contribute to tumorigenesis by suppressing senescence and promoting the cell cycle, further studies are needed to fully elucidate the processes by which the YAP/TEAD complex regulates senescence and tumorigenesis.

### 2.3. Kinase Activity

The core of the Hippo pathway is a kinase cascade. In this cascade, Mst1/2 kinases and SAV1 form a complex that phosphorylates and activates LATS1/2. The LATS1/2 kinase then phosphorylates YAP/TAZ, inhibiting its activity and suppressing cell proliferation.

The inhibition of the activity of the tumor suppressor MST1 induces senescence. Alveolar rhabdomyosarcoma is a sarcoma characterized by the expression of the paired box 3-forkhead box protein O1 (PAX3-FOXO1) fusion oncogene. The up-regulation of Ras-association domain family (RASSF) 4, a member of RASSF, by PAX3-FOXO1, inhibited MST1 activity, which induced senescence avoidance and promoted cancer through cell cycle progression [46]. Furthermore, under oxidative stress, MST1 was found to phosphorylate and activate the transcription factor FOXO1/3a [47]. FOXO3a has been shown to cause cellular senescence via cell cycle arrest in the G2/M phase by inducing the expression of growth arrest and DNA damage-inducible protein 45α [48]. MST1 has been suggested to inhibit cell proliferation via cellular senescence by transcriptionally activating the FOXO family and regulating the cell cycle. Furthermore, the increased expression and activation of MST1 were shown to play a role in foamy macrophage senescence [49]. The MST1-mediated induction of p53, p21, and p16 expression may induce macrophage senescence and aggravate atherosclerosis. The Hippo pathway may be closely involved in the development of age-related diseases and, thus, the regulation of senescent cells by its core kinases, MST1/2, warrants further investigation.

In hepatocytes, the loss of LATS1/2 resulted in senescent dysplastic hepatocytes [38]. The hyperactivation of YAP/TAZ by the deletion of LATS1/2 induced oncogenic stress in cells, which was assumed to damage DNA and activate p53. The suppression of YAP over-activation by LATS1/2 may inhibit p53-dependent senescence and apoptosis in senescence-prone cells. A previous study demonstrated that elevated intracellular ROS levels inhibited cell division by reducing LATS1 levels, causing stable cell cycle arrest in aging cells [50]. AMPK-associated protein kinase 5 (NUAK1), the expression of which increases during early senescence, has been reported to phosphorylate LATS1 at Ser464 and inhibit its stabilization [51]. Oxidative stress induced by ROS was also found to activate NUAK1 and contributed to the formation of colorectal cancer [52]. Therefore, the regulation of the NUAK1-LATS1 axis by oxidative stress may be important for controlling premature cellular senescence.

However, LATS2 has been reported to function cooperatively with retinoblastoma protein (RB), a cancer suppressor that induces cellular senescence [53]. The LATS2 locus is physically linked to RB on 13q and acts synergistically at the transcription factor E2F regulatory level. LATS2 promotes the complete silencing of *E2F* target genes and the induction of senescence-like states by RB. When cells are exposed to DNA damage stress, functional cooperation between RB and LAT2 is an important factor in inducing cellular senescence and suppressing tumor development.

Furthermore, the Hippo pathway has been implicated in aging. Intervertebral disc degeneration (IVDD), a cause of back pain in the elderly, is closely related to the aging of nucleus pulposus cells. The mechanisms underlying this senescence involve the phosphorylation of MST1/2 by the abnormal activation of the Hippo pathway, which induces the phosphorylation of LAT1/2 and suppresses the activity of YAP/TAZ, thereby activating p53 [54]. The iridoid glycoside morroniside has been shown to promote cellular anti-aging and alleviate the progression of IVDD by inhibiting the phosphorylation of MST1/2. Further studies on the crosstalk between the Hippo pathway and aging may provide further insights into aging.

## 3. The Hippo Pathway and Cellular Senescence in Tissue Remodeling

The Hippo pathway regulates the development and size of organs and plays an important role in tissue homeostasis. However, cellular senescence is involved in many physiological processes that require tissue remodeling. In this Section, we describe the functions of senescent cells that are regulated by the Hippo pathway in tissue homeostasis (Table 1).

### 3.1. Embryogenesis

Cellular senescence is tightly regulated and plays an important role in mammalian embryogenesis [55,56]. Senescent cells expressing SA-β-gal and Ki-67 are present across various regions of the developing embryo, including the ectoderm and endolymphatic sac. During development, senescent cells coordinate normal limb formation and tissue remodeling by inducing apoptosis or being eliminated by macrophages. Developmental senescent cells express p21, but not p53 or p16. SA-β-gal cells are lost in p21-deficient embryos, but not in p53- or p16-deficient embryos. This up-regulation of p21 is independent of p53 and is mediated by TGF-β/Smad and phosphoinositide 3-kinase (PI3K)/FOXO signaling. Various intracellular signaling pathways precisely regulate embryonic development, and the differential activity of the Hippo pathway in blastocysts affects cell polarity and advances development. In the outer epithelial ectoderm, the Hippo pathway is inactivated and TAZ is activated. A previous study demonstrated that TAZ bound to Smad and promoted its nuclear translocation under a TGF-β stimulation [57]. It also promoted the activation of PI3K signaling. During embryonic development, the activation of TAZ via the inactivation of the Hippo pathway has been suggested to promote the activation of TGF-β and PI3K signaling, thereby regulating senescent cells in the embryo.

### 3.2. Fibrosis

The fibrotic response is activated during the repair process and forms excessive amounts of connective tissue. The accumulation of extracellular matrix (ECM) proteins in tissue has been shown to induce permanent structural changes and dysfunction [58]. Cellular senescence plays a facilitatory or obstructive role in this process.

During skin wound healing, elevated nuclear YAP/TAZ levels have been detected in the dermis. The decreased expression of YAP/TAZ reduced the expression levels of TGF-β and its target genes, p21 and Smad family members, and delayed healing [59]. The induction of p21 expression by YAP/TAZ resulted in the senescence of skin fibroblasts [60], which secrete anti-fibrotic MMPs, degrade ECM components, and suppress fibrosis.

In the liver, chronic tissue damage leads to excessive fibrosis, which decreases organ function and ultimately results in liver failure. Common injuries to the liver are hepatitis virus infection, excessive alcohol consumption, and nonalcoholic steatohepatitis (NASH). These damaging stimuli induce hepatic stellate cells (HSCs) to differentiate into myofibroblasts that produce ECM [61]. In addition, HSCs, mediators of liver fibrosis, are rapidly activated and transformed into myofibroblasts upon liver injury. In mice treated with CCl_4_, fibrosis caused by liver injury was associated with an increase in the number of senescent HSCs. These senescent HSCs resolved fibrosis by decreasing the production of ECM components and increasing the expression of anti-fibrotic SASP factors, such as proteases and MMPs [62]. Senescence was also shown to be induced by IL-22 [63], which promoted HSC senescence in a p53-dependent manner via STAT3 and SOCS3 as well as IGF-1 [64]. On the other hand, YAP was found to predominantly localize in the nucleus of HSCs in CCl_4_-induced mouse liver fibrosis models and in the liver of hepatitis C virus-infected patients [65]. YAP is known to interact with STAT3 and suppress its activity [66],which inhibits the production of IL-22 and IGF-1. Therefore, the activation of YAP in HSCs is expected to suppress senescence-induced fibrosis. A previous study demonstrated that the inhibition of YAP by siRNA or of the YAP/TAZ-TEAD interaction decreased target gene expression and prevented the transdifferentiation of quiescent HSCs into myofibroblasts [67]. TAZ expression was found to be up-regulated in the livers of NASH patients and in hepatocytes from NASH model mice [68]. The induction of senescent HSCs due to the activation of the Hippo pathway may be an effective target for the treatment of lesions of liver fibrosis.

Idiopathic pulmonary fibrosis (IPF) is a chronic lung disease characterized by decreased lung function, and risk factors include smoking and exposure to environmental toxins [69]. Senescence biomarkers, such as Ki-67 and p16, have been detected in human IPF, suggesting a pathological role for aging in this disease [70,71]. In contrast to other fibrotic lesions, the accumulation and persistence of pulmonary senescence exacerbates pulmonary fibrosis. Senescent lung fibroblasts induce myofibroblast differentiation in a paracrine manner, suggesting that they express profibrotic SASP [71]. Pulmonary senescence is mediated by increases in NADPH oxidase 4 (NOX4) and decreases in NFE2-related factor 2 (Nrf2). Therefore, ROS levels increase, leading to DNA damage and senescence [70]. YAP and TAZ were previously shown to be expressed in the nucleus and promoted fibrosis in the lung foci of IPF patients [72]. In lung fibroblasts, TAZ/YAP were found to function cooperatively with TGF-β, a central mediator of lung fibrosis [72,73]. TGF-β activates NOX4 [74] and Nrf2 inhibits TGF-β signaling [75]. In pulmonary fibroblasts, the activation of TAZ/YAP has been suggested to contribute to fibrosis by activating TGF-β signaling and promoting cellular senescence via oxidative stress. On the other hand, TGF-β has been reported to induce the expression of the senescence-regulating factor P300/CBP-associated factor (FRMD6) [76]. In fibroblasts, FRMD6 promoted cellular senescence by activating MST kinase and inactivating YAP/TAZ. Further studies are needed on the effects of the interactions between TGF-β signaling and YAP/TAZ in IPF, with a focus on the relevant regulatory factors.

### 3.3. Tissue Reprogramming

Differentiated somatic fibroblasts are reprogrammed into multipotent cells by the expression of four Yamanaka factors (OCT3/4, SOX2, c-Myc, and KLF4) in vitro. However, the efficiency of this process is very low, suggesting that cellular senescence is a barrier to reprogramming [77,78]. In mouse and human fibroblasts, the expression of the four factors was shown to activate cellular senescence markers, such as SA-β-gal. The induction of senescence by the four factors contributes to reprogramming via the activation of p16 and p21. c-Myc and KLF4 also contribute to p21 expression via p53 [79]. Previous studies have demonstrated that mouse and human fibroblasts lacking p21 or p53 generated more induced pluripotent stem cell colonies, suggesting that the suppression of senescence increases the efficacy of reprogramming [79,80,81]. The activation of Hippo-YAP signaling also efficiently induced tissue reprogramming [82]. Moreover, YAP/TAZ were found to suppress p53 activity and inhibit cellular senescence [30,83]. In tissue reprogramming, the efficient activation of the Hippo pathway may suppress barriers to senescence. Further studies on the crosstalk between the Hippo pathway and cellular senescence will lead to the development of regenerative medicine.

### 3.4. Stem Cells

Stem cell senescence reduces the capacity for self-renewal and differentiation, limiting tissue regeneration and healing. Hematopoietic stem cells contribute to hematopoietic homeostasis and regeneration after injury through self-renewal, proliferation, and differentiation. Hematopoietic stem cells may activate NOX and produce ROS by various external and internal stresses. ROS activate the p53-p21 pathway by inducing DNA double-strand breaks and Hematopoietic stem cell senescence [84]. The regeneration of the epidermis and mucosal epithelium is also highly dependent on resident epithelial stem cells (ESCs). The inhibition of mTOR with rapamycin has been shown to increase the self-renewal potential of the oral mucosa by preventing ESC senescence [85]. The Hippo signaling pathway plays an important role in maintaining tissue homeostasis and sizes by regulating tissue-specific stem cells. In epithelial ovarian cancer, YAP/TAZ have been shown to activate NOX2 [86]. YAP/TAZ also activate mTOR [87]. The suppression of the Hippo pathway and the activation of YAP/TAZ may act on the stem cells of tissues in a senescence-promoting manner, inhibiting efficient regeneration and tissue homeostasis. Therefore, the proper control of senescence regulation in stem cells by the Hippo pathway may help advance regenerative medicine.

Epigenetics plays an important role in aging and senescence and has been reported as a hallmark of the aging process [88]. Epigenetic changes over time in tissue stem cells contribute to stem cell depletion and aging. DNA methylation is a reaction in which a methyl group is added to the cytosine of a CpG dinucleotide and is catalyzed by DNA methyltransferases (DNMTs), which repressively regulate gene expression. A recent study reported that the DNA methylation of stem cells regulated their division into stem cells or non-stem cells [89]. Histone demethylases have also been shown to inhibit Hematopoietic stem cell senescence in a methylation activity-dependent manner [90]. YAP/TAZ have been reported to contribute to DNA methylation by activating DNMT1 and by interacting with DNMT3 [91,92,93]. Hippo pathway-related factors are expected to have an impact on epigenetic changes in stem cell senescence; however, limited information is currently available. Further studies on the effects of the Hippo pathway on senescence and epigenetic changes in different tissue stem cells will contribute to the development and clinical application of rejuvenation research targeting the epigenome.

**Table 1:** Hippo pathway-related factors in tissue remodeling and senescence.

Tissue Remodeling	Signaling Factor	Regulators	Trend	Positive/Negative ^2^	Ref
Embryogenesis	YAP/TAZ	TGF-β, PI3K	Senescence	Positive	[57]
Fibrosis	YAP/TAZ	TGF-β (skin)	Senescence	Negative	[59,60]
	YAP	STAT3 (liver)	Anti-senescence	Negative	[64]
Tissue reprogramming	YAP/TAZ	p53	Anti-senescence	Positive	[82]
Stem cells	YAP/TAZ	NOX, mTOR	Senescence	Negative	[86,87]
	YAP/TAZ	DNMT	N/A ^1^	N/A	[91,92,93]

^1^ N/A, no data were available. ^2^ Positive/Negative, effects on tissue remodeling.

## 4. The Hippo Pathway and Cellular Senescence in Cancer

**Figure 2:**
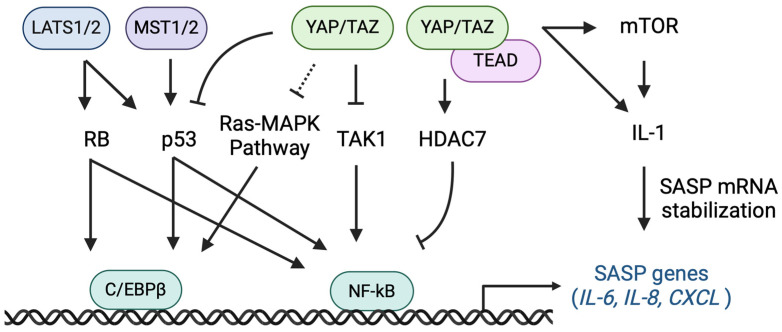
Regulation of SASP factor expression by the Hippo pathway [23,25,94,95,96,97,98,104,105,106,107,108,109,110,111,112,113,114,115,117,121,126,127]. LATS1/2, MST 1/2, and YAP/TAZ regulate the expression of several interleukins and chemokines, which are SASP factors, via the transcription factors C/EBPβ and NF-κB. Hippo pathway-associated factors contribute to cellular senescence by regulating the expression of SASP factors through a number of molecules. Arrows and vertical bars indicate potentiating and inhibitory effects. Dashed lines indicate potential.

Cellular senescence is an important inhibitory mechanism for cancer as a potent barrier to tumorigenesis because it suppresses excessive cell division in normal cells. However, inflammation-inducing SASP factors secreted by senescent cells have been implicated in various inflammatory diseases, including cancer. While senescence suppresses tumor formation, it has also been shown to play a role in tumorigenesis, including tumor initiation, progression, invasion, and metastasis. This Section discusses therapeutic applications for aging cancer cells, with a focus on the crosstalk between SASP regulators and the Hippo pathway, which positively and negatively regulate tumors (Figure 2).

### 4.1. SASP-Associated Transcription Factors and the Hippo Pathway

In mammalian cells, the activation of oncogenes induces proliferative stress and senescence, which inhibits tumor growth. Therefore, senescence is a physiological mechanism for tumor suppression that inhibits the development of malignant tumors due to excessive cell proliferation. The transcription factors CCAAT/enhancer binding-protein-β (C/EBPβ) and nuclear factor-κB (NF-κB) are specifically activated in senescent cells. These transcription factors play an important role in the expression of SASP factors.

NF-κB is a transcription factor that regulates the expression of genes for chemokines that bind to the chemokine receptor, C-X-C Motif Chemokine Receptor 2 (CXCR2) [23,25]. A proteomic analysis of proteins that specifically bind to the chromatin of senescent cells identified the NF-κB subunit p65 as a major transcription factor that accumulates on the chromatin of senescent cells [94]. Cellular senescence signals, such as the activation of Ras, induce the phosphorylation of Serine 536 of p65, which translocates NF-κB into the nucleus and induces the expression of SASP factors, including IL-6 and CXCL. As a master regulator of SASPs, NF-κB affects the expression of many genes. YAP/TAZ, downstream factors of the Hippo pathway, suppress NF-kB activity. YAP has been shown to interact with TRAF6, promoting its degradation by polyubiquitination and weakening the TRAF6-mediated activation of NF-kB [95]. The expression of NF-κB, a proinflammatory cytokine IL-6, tumor necrosis factor-α (TNF-α), and IL-1β was up-regulated in endothelial-specific YAP knockout mice in response to LPS. Furthermore, the overexpression of YAP inhibited TGF-β-activated kinase 1 (TAK1) activity and attenuated the activation of downstream NF-κB signaling, thereby down-regulating the expression of proinflammatory cytokines, such as TNF-α, IL-1β, and IL-6 [96]. When activated YAP/TAZ translocate into the nucleus, it forms a complex with the transcription factors TEADs. This YAP/TAZ-TEAD complex recruits histone deacetylase 7 to the promoter region of NF-κB target genes and may reduce the production of inflammatory cytokines [97]. In tumors, senescence signaling by Ras/MAPK was found to suppress YAP/TAZ activity [40]. Senescent tumors inhibit YAP/TAZ activity, induce SASP factor expression by promoting NF-κB activation, and eliminate tumor cells by activating immunity.

C/EBPβ is activated by the Ras-MAPK pathway, which is involved in cell proliferation, differentiation, and cell death. C/EBPβ functions homeostatically in senescent cells, directly regulating the expression of IL-6, a typical SASP factor, and inducing cellular senescence. The Hippo pathway shares many targets with the Ras-MAPK pathway and regulates cancer cell growth and cell death through a crosstalk mechanism [98]. Therefore, the simultaneous activation of the Ras-MAPK pathway and Hippo pathway exerts synergistic effects and is effective in cancer therapy. For example, in T-ALL cells and breast cancer cells, vitamin E analogs were shown to activate MST1 and ERK signaling, which induced apoptosis [99]. In osteosarcoma cells, the flavonol fisetin activated LATS and ERK kinases and induced apoptosis [100]. Curcumin, a polyphenolic compound, induced cell cycle arrest via the generation of ROS, the activation of ERK and MST kinases, and the down-regulation of YAP proteins in various cancer cell models [101,102,103]. Crosstalk between the Ras-MAPK pathway and the Hippo pathway may inhibit cancer malignancy through the regulation of cellular senescence. Conversely, the inactivation of the Hippo pathway increased IL-6 expression in the mouse liver [104], suggesting that the Hippo pathway regulates the Ras-MAPK pathway and may control the activity of C/EBPβ; however, this crosstalk needs to be examined in more detail.

Although there are many factors that induce senescence, including telomere dysfunction, chemotherapeutic agents, DNA damage, and oncogene expression, the molecular pathways that activate and enforce senescence converge on the p53 and Rb tumor suppressor pathways [105,106,107]. The induction of p21 expression by the activation of p53 or the activation of Rb by the expression of p16, a cyclin-dependent inhibitor, is required for cell cycle arrest. Following the induction of cell cycle arrest by the activation of the p53 and Rb pathways, many downstream effectors, such as p38-MAPK, NF-κB, and C/EBPβ, are activated, leading to senescence. In senescent cancer cells, the persistent expression of SASP is considered to exert various physiological effects on tumors by promoting cell cycle arrest and senescence. The Hippo pathway affects the activity of p53, which is important in cellular senescence. MST1, a core kinase of the Hippo pathway, inhibits p53 deacetylation by SIRT1 and activates p53 [108]. LATS1 has also been shown to induce p53 in response to the activation of K-RAS [109]. LATS2 activates p53 by phosphorylating and activating the pro-apoptotic factor, apoptosis-stimulating protein of p53 [110]. Furthermore, LATS1/2 suppressed the activity of MDM2, which induced the degradation of p53 [109,111]. On the other hand, the loss of YAP/TAZ induced the activation of p53 [83,112,113]. We previously reported that TAZ induced the deacetylation of p53 and suppressed its DNA-binding ability, thereby inhibiting the ability of p53 to activate transcription [114]. The activation of p53 by the knockdown of TAZ resulted in cellular senescence by inducing the expression of p21. Hippo signaling in cancer cells has been suggested to activate p53 by phosphorylating MST1/2 and LATS1/2 and inhibiting YAP/TAZ, coordinately promoting cellular senescence and the secretion of SASP factors. Conversely, the inhibition of the Hippo pathway in cancer cells induces the activation of YAP/TAZ, which suppresses cellular senescence.

### 4.2. SASP-Related Signaling Factors and the Hippo Pathway

IL-1 mRNA is highly expressed in senescent fibroblasts, and the effects of IL-1 on SASP factors have attracted much attention. The binding of IL-1α to IL-1 receptor 1 promotes the activation of IL-1 receptor-associated kinase 1 and also activates NF-κB, which induces the expression of SASP genes. The inhibition of IL-1α expression was found to strongly suppress the gene expression of the SASP factors IL-6 and IL-8, suggesting that IL-1α is a master regulator that acts upstream of SASP factors [115]. In addition, the activation of IL-1β has been shown to induce SASPs in hepatic astrocytes, indicating that IL-1α and IL-1β are used in different manners by various tissues to induce senescence [116]. In malignant mesothelioma (MM), TAZ, a downstream factor of the Hippo pathway, was shown to regulate IL-1 signaling activity and promote downstream gene expression [117]. The knockdown of IL-1β and application of IL-1 receptor antagonists significantly suppressed the malignant phenotype of immortalized mesothelial cells and MM cells activated by TAZ. In MM, TAZ has been shown to promote the transcription of cytokine-related genes, including *IL-1β*, and the IL-1β-induced proliferation of senescent MM cells. TAZ also regulates IL-1 signaling activity and induces the expression of SASP factors, which may be involved in senescence.

mTOR, a regulator of cell growth and metabolism, is a serine/threonine kinase that senses diverse environmental signals and is activated via the PI3K-AKT pathway [118,119]. The promotion of IL-1α translation by activated mTOR induces SASP factor expression by activating the transcription of NF-kB, which promotes tumor formation [120]. YAP, a downstream target of the Hippo pathway, is an important mediator in the regulation of mTOR. YAP down-regulates PTEN by inducing miR-29 and inhibiting PTEN translation [87]. Since PTEN normally suppresses PI3K activity, YAP regulates cell size, tissue growth, and hyperplasia through its activation of PI3K-mTOR. A previous study reported that YAP manipulated the growth of lung adenocarcinoma, which was regulated by PTEN/AKT/mTOR autophagy signaling [121]. Therefore, YAP may promote tumorigenesis by inducing the expression of SASP factors through the activation of the mTOR pathway.

The p38-MAPK pathway activates tumor suppressor pathways, such as p53/p21 and p16INK4A, which are essential for the suppression of cancer through cell growth arrest in cellular senescence [122]. On the other hand, p38 has been reported to inhibit the Hippo pathway and activate YAP by suppressing LATS1/2 activity. The p38-induced activation of YAP is oriented toward cellular senescence by inducing SASP factors and promoting tumor formation [123]. Therefore, the p38-MAPK-mediated regulation of tumor formation warrants further study to clarify these conflicting effects.

### 4.3. Oncogene-Mediated Cellular Senescence and the Hippo Pathway

The activation of oncogenes leads to proliferative stress and the induction of senescence, which limits tumor growth. A previous study demonstrated that the activation of the oncogene RAS alone was not sufficient to promote cellular oncogenesis, and tumor growth was prevented by the induction of senescence [124]. Tumor growth requires additional hits, such as the activation of oncogenes and the inactivation of tumor suppressors, and the activation of Myc, E1A, and DRIL1 has been demonstrated. Myc is a target gene of YAP/TAZ, and its expression is regulated at the transcriptional and post-transcriptional levels [125]. In basal cell carcinomas of the skin, Myc and target genes of the Hippo-YAP pathway are up-regulated, and YAP/TAZ and RAS activation are jointly involved in cell growth [126,127]. The presence of a mutant p53 protein is also a hit factor that contributes to the avoidance of oncogene activation-induced senescence. p53 mutants are found in more than 50% of human cancers. YAP has been shown to physically interact with p53 mutants and positively regulate the expression of cyclin A, cyclin C, and CDK1, leading to cancer cell proliferation [128]. The activation of YAP/TAZ via the inactivation of the Hippo pathway may enhance hit factors that promote evasion from the aging mechanism, which acts as a barrier to the malignant transformation of cancer.

### 4.4. Treatment-Induced Senescence and the Hippo Pathway

Anticancer agents, such as doxorubicin, cisplatin, etoposide, docetaxel, and vincristine, force some tumor cells into senescence [129]. Doxorubicin was shown to induce p53-dependent senescence as well as senescence in cells lacking p53 and p21, and also down-regulated YAP expression through the phosphorylation of MST1/2 and LATS1 [129]. The inhibition of senescence and the up-regulated expression of YAP/TAZ have been reported in cisplatin- and docetaxel-resistant cancer cells [130,131]. These findings suggest the involvement of the Hippo-YAP/TAZ pathway in the induction of cellular senescence by cancer drugs and drug resistance through the inhibition of senescence. Further studies on the regulation of senescence by the Hippo pathway may be useful for enhancing the therapeutic effects of existing agents.

## 5. The Hippo Pathway and Cellular Senescence in Diseases

**Figure 3:**
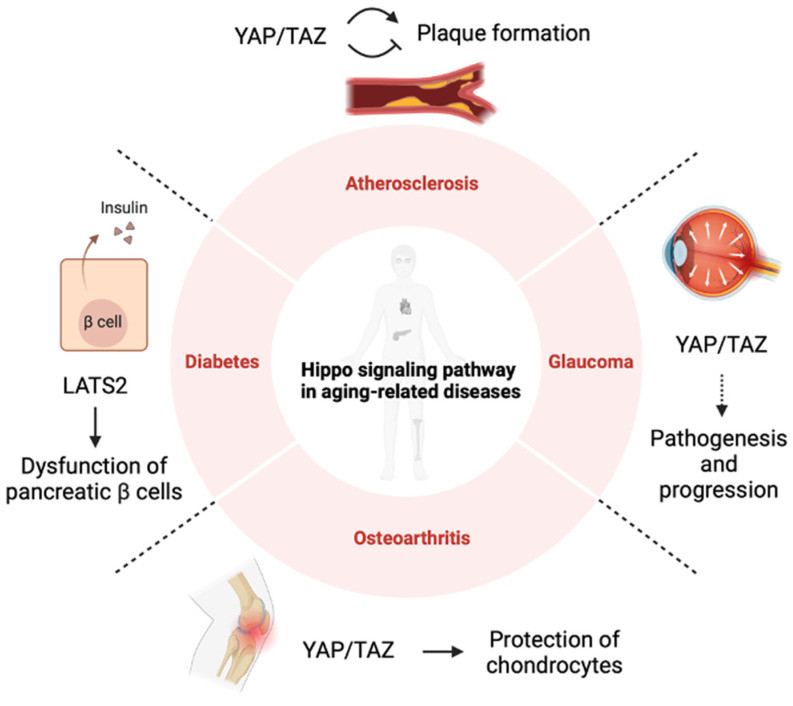
Relationship between the Hippo pathway and aging-related diseases [57,120,135,136,140,146,147,148,150]. The Hippo pathway is involved in physical aging by regulating cellular senescence in tissues. Arrows indicate potentiation and dashed lines indicate potential.

Cells have a number of fates in response to various stimuli and stresses, such as differentiation, proliferation, senescence, and death. Stress from the onset of diseases associated with physical aging and the persistence of chronic symptoms are closely related to cellular senescence and have been shown to play a role in lifespan. This Section describes the effects of factors of the Hippo pathway on senescence-related diseases and cellular aging (Figure 3).

### 5.1. Atherosclerosis

Atherosclerosis develops due to the foaming and accumulation of macrophages induced by the inflammatory response of activated endothelial cells and vascular smooth muscle cells. Human atherosclerotic plaques have been reported to contain senescent endothelial cells and vascular smooth muscle [132,133]. Senescent cells that form plaques are protective against atherosclerosis. Conversely, SASP factors secreted by senescent cells that are not removed and accumulate contribute to symptom progression [134]. In endothelial cells and vascular smooth muscle cells that comprise atherosclerotic plaques, shear stress caused by changes in blood flow is known to activate YAP/TAZ. YAP/TAZ were previously shown to activate JNK signaling, which is important for the inflammatory response, and induced the expression of IL-6 and IL-8, key inflammatory cytokines for SASP [135]. Furthermore, the knockdown of YAP in endothelial cells delayed plaque formation. These findings suggest that the activation of YAP/TAZ in senescent endothelial cells and vascular smooth muscle cells in atherosclerosis induces the expression of SASP factors and exacerbates plaque formation. Therapies that target YAP/TAZ to eliminate senescent cells may be used for the prevention or treatment of atherosclerosis. In addition, the expression of PCAF, which is involved in atherosclerotic plaque and neointima formation, was found to be up-regulated in the aortic endothelial cells of aging mice. PCAF has been suggested to suppress YAP/TAZ activity via the Hippo pathway and regulate endothelial cell senescence [136]. The tight regulation of YAP/TAZ activation levels may be a target for the prevention and treatment of atherosclerosis and, thus, warrants further research.

### 5.2. Osteoarthritis

Osteoarthritis (OA) is a disease in which an inflammatory response in the chondrocytes of the joints induces progressive degeneration and an age-dependent loss in the ability to maintain cartilage tissue [137]. It is one of the factors causing chronic pain and mobility issues in the joints of the elderly. Chondrocytes from OA joints have been shown to express various senescence markers, such as SA-β-gal and p16 [138]. Furthermore, in experiments on mice, the removal of senescent cells was an effective treatment for OA [139]. In mouse models of OA, the activation of YAP has been suggested to protect chondrocytes and to be necessary for the maintenance of articular cartilage homeostasis. The inflammatory cytokines TNF-α and IL-1β secreted by OA chondrocytes were found to induce the degradation of YAP/TAZ via the activation of TAK1, which promoted damage to cartilage [140]. Senescent chondrocytes in OA may suppress the expression of YAP/TAZ by pro-inflammatory SASP factors. Therapies that selectively eliminate senescent cells by targeting the activation of the Hippo pathway and YAP/TAZ expression in chondrocytes may effectively prevent and treat OA.

### 5.3. Glaucoma

Glaucoma, the leading cause of blindness worldwide, is caused by the degeneration of the optic nerve. The most common form of glaucoma is primary open-angle glaucoma (POAG), for which an advanced age is one of the main risk factors [141]. POAG is characterized by increased intraocular pressure (IOP), which is considered to cause retinal ganglion cell death. Elevated IOP was previously shown to increase the transcription of p16 and induce the senescence of retinal ganglion cells [142]. Therefore, further studies are needed to clarify whether a relationship exists between the development of cellular senescence and glaucoma.

The activation of the TGF-β/Smad signaling pathway in the human trabecular retina (HTM) promotes the synthesis of collagen and inhibits that of hyaluronic acid, thereby promoting the accumulation of ECM components. These changes ultimately increase aqueous outflow resistance and IOP, resulting in POAG [143,144]. TGF-β also arrests the cell cycle by inducing the expression of the cyclin-dependent kinase inhibitors p15, p21, and p27 and by suppressing that of several growth factors, including c-Myc [145]. TGF-β has been shown to induce or promote senescence and age-related features in various cell types and may also contribute to the senescence of retinal ganglion cells [146,147].

A previous study demonstrated that TAZ bound to the Smad2/3/4 complex and was recruited to TGFβ response elements in response to a TGF-β stimulation [57]. TAZ is a mediator of the nucleocytoplasmic localization of Smad and in its presence, the Smad2/3/4 complex accumulates in the nucleus and activates transcription. In glaucoma, the control of TAZ activity by the Hippo pathway contributes to TGF-β signaling-mediated ganglion cell senescence and cell death, which may play a role in disease onset and progression. YAP is important for the maintenance of normal HTM; however, TAZ has also been reported to significantly affect HTM in glaucoma [148]. The role of the Hippo pathway in the relationship between the onset of cellular senescence and glaucoma requires further study.

### 5.4. Type 2 Diabetes (T2DM)

Aging is a risk factor for T2DM, the onset of which is caused by a decline in the insulin-secreting function of pancreatic beta cells. Cellular senescence has been reported in the islet cells and adipocytes of patients with T2DM. The increased expression of p16 in the islets of old mice was shown to suppress islet cell proliferation [149]. Furthermore, under diabetic conditions, the Hippo signaling component LATS2 is activated and induces the apoptosis and dysfunction of pancreatic beta cells by activating the mechanistic target of rapamycin complex 1 (mTORC1), a physiological inhibitor of autophagy [150]. Autophagy controls intracellular quality by degrading intracellular components and removing abnormal protein aggregates and organelles. The inhibition of mTOR signaling has been suggested to exert anti-aging effects and prolong lifespan because it acts to protect cells. Furthermore, mTOR signaling promoted the production of inflammatory SASP factors via IL-1α and NF-κB [120]. The LATS2-mTORC1 activation axis in T2DM may contribute to the deterioration of cellular functions and aggravation of symptoms by promoting senescence and a sustained inflammatory response in pancreatic beta cells. Detailed investigations on the mechanisms responsible for the activation of the Hippo pathway under cellular stress, as well as the regulatory mechanisms of senescence in chronic T2DM, may lead to the prevention and treatment of age-related diabetes.

## 6. The Hippo Pathway and Senotherapy

Senolytics, which eliminate senescent cells, and senomorphics, which prevent the harmful effects of senescent cells, have attracted attention as ‘senotherapies’ for the treatment of aging. The development of compounds with pharmacological effects, such as senolytics and senomorphics, is underway, while the repositioning of existing drugs is also receiving attention. Navitoclax, in phase 3 clinical trials on myelofibrosis, inhibited the activity of BCL-2 family members and promoted the apoptosis of senescent cells [151]. The combination of the tyrosine kinase inhibitor dasatinib with the flavonols quercetin and fisetin, which target the PI3K/AKT/mTOR pathway, has also been used in clinical trials to eliminate senescent cells [152,153]. As an alternative to dasatinib, natural senescence inducers have been identified, such as piperlongumine from long peppers and parthenolide and curcumin from various edible plants [154]. Fisetin and curcumin activate LATS and MST and down-regulate the expression of YAP [100,101,102,103]. Combinations of these compounds with Hippo pathway activators and YAP/TAZ inhibitors may be used as potent senotherapeutic agents. YAP/TAZ are involved in the activation of the BCL2 and PI3K pathways and, thus, their inhibition may be an effective strategy for the elimination of senescence cells through multiple pathways.

Senomorphic agents inhibit SASP production and secretion by senescent cells and prevent growth arrest [155]. The mTOR inhibitor rapamycin and its analog RAD001 attenuated SASP production and prevented senescence in spontaneously aging mice [156,157]. Metformin and the naturally occurring flavonoids apigenin and kaempferol were shown to inhibit NF-κB signaling and reduce SASP production [158,159]. The inhibition of Hippo pathway activity and the activation of YAP/TAZ suppressed the activation of the NF-κB pathway, thereby reducing the expression of SASP factors. However, the activation of YAP/TAZ may also affect that of mTOR. The combined use of YAP/TAZ inhibitors with rapamycin and flavonoids may effectively inhibit the inflammatory properties of aging cells and delay the onset and progression of aging and age-related diseases. Since Hippo pathway-related factors exert opposite effects on senolytic and senomorphic activities, Hippo pathway activators and inhibitors need to be used with careful consideration of the extent of age-related disease progression and the state of aging cells. Further research into the regulation of cellular aging by the Hippo pathway will clarify its use and utility as a senotherapeutic agent.

## 7. Conclusions

Cellular senescence is the process by which cells stop proliferating in response to various stresses and remain in a stable state, contributing to the maintenance of the individual and homeostasis. The control of cellular senescence has implications for disease development, treatment, and even lifespan. In vivo studies demonstrated that cellular senescence acts as a potent tumor suppressor. Although not currently being developed as senescence-inducing drugs, a number of recent targeted therapeutics involve the senescence response as part of their effects [160]. New small-molecule compounds, such as p53 activators, CDK inhibitors, Myc inhibitors, and PTEN inhibitors, have progressed to preclinical or clinical trials [160,161,162,163]. Cancer treatment is expected to combine approaches targeting the promotion of senescence with conventional therapies, which will increase the efficacy of both. Components of the Hippo pathway have been extensively implicated in the regulation of senescence-related signaling pathways. Antisense nucleotide and TEAD inhibitors targeting the Hippo pathway and YAP mRNA are currently being introduced into clinical practice for the treatment of MM and advanced solid tumors [164]. Senescence-inducing drugs and Hippo pathway-targeting drugs have the potential to exert unprecedentedly powerful synergistic effects and are expected to be introduced into clinical settings.

Multiple pathways are involved in senescence, including DDRs, epigenetic regulation, and oxidative stress pathways. As basic research on senescence progresses, an integrated understanding of the interactions of complex intracellular pathways will be developed. In addition, comprehensive analyses, such as single-cell RNA sequencing and proteome analyses, will reveal new markers of senescence and the molecular mechanisms involved in the dynamics of the progressive stages. Further studies on the crosstalk between novel molecular mechanisms and Hippo pathway-related factors will provide a more detailed understanding of the reversibility of aging and the long-term effects of SASPs, as well as fundamental insights into the aging process in organizations and individuals as a whole. Clinical applications will focus on the development of strategies to prevent and treat age-related diseases. The development of drugs such as senolytics and senomorphics that specifically target senescent cells and their deleterious effects is expected, with the aim of reducing side effects. Advances in drug development and contributions to personalized medicine through the establishment of biomarkers will lead to an increase in healthy life expectancy in the future. As noted herein, advances in research focusing on the Hippo pathway and senescence may clarify the mechanisms underlying the regulation of cellular senescence by Hippo pathway-related factors and the establishment of new biomarkers and therapeutic targets for age-related diseases, leading to advances in the study of aging.

Data generation in biology and biotechnology has markedly increased in recent years due to the rapid development of high-performance technologies [165]. Hippo pathway-related factors have also been shown to crosstalk with various intracellular pathways using machine learning to evaluate transcriptional profiles [166]. In the future, further details on the crosstalk between aging and the Hippo pathway will be obtained by using artificial intelligence to analyze the vast amount of data generated in the genome field and biology.

Recent findings highlight the unexpected disadvantages of cellular senescence. SASP factors secreted by senescent cells that accumulate with age have been implicated in the development of a wide range of diseases, including carcinogenesis, atherosclerosis, dementia, and endocrinopathies. Future research to investigate the regulatory mechanisms underlying cellular senescence by the Hippo pathway is expected, which will also lead to analyses of the mechanisms responsible for the appropriate clearance of senescent cells. The development of drugs that induce senescence or remove senescent cells via the Hippo pathway may contribute to innovative treatments for age-related diseases.

## Data Availability

Not applicable.
